# Classification and assessment tools for structural motif discovery algorithms

**DOI:** 10.1186/1471-2105-14-S9-S4

**Published:** 2013-06-28

**Authors:** Ghada Badr, Isra Al-Turaiki, Hassan Mathkour

**Affiliations:** 1 King Saud University, College of Computer and Information Sciences, Riyadh, Kingdom of Saudi Arabia; 2 IRI - The City of Scientific Research and Technological Applications, University and Research District, P.O. 21934 New Borg Alarab, Alexandria, Egypt

## Abstract

**Background:**

Motif discovery is the problem of finding recurring patterns in biological data. Patterns can be sequential, mainly when discovered in DNA sequences. They can also be structural (e.g. when discovering RNA motifs). Finding common structural patterns helps to gain a better understanding of the mechanism of action (e.g. post-transcriptional regulation). Unlike DNA motifs, which are sequentially conserved, RNA motifs exhibit conservation in structure, which may be common even if the sequences are different. Over the past few years, hundreds of algorithms have been developed to solve the sequential motif discovery problem, while less work has been done for the structural case.

**Methods:**

In this paper, we survey, classify, and compare different algorithms that solve the structural motif discovery problem, where the underlying sequences may be different. We highlight their strengths and weaknesses. We start by proposing a benchmark dataset and a measurement tool that can be used to evaluate different motif discovery approaches. Then, we proceed by proposing our experimental setup. Finally, results are obtained using the proposed benchmark to compare available tools. To the best of our knowledge, this is the first attempt to compare tools solely designed for structural motif discovery.

**Results:**

Results show that the accuracy of discovered motifs is relatively low. The results also suggest a complementary behavior among tools where some tools perform well on simple structures, while other tools are better for complex structures.

**Conclusions:**

We have classified and evaluated the performance of available structural motif discovery tools. In addition, we have proposed a benchmark dataset with tools that can be used to evaluate newly developed tools.

## Introduction

Finding recurring patterns, motifs, in biological data gives an indication of important functional or structural roles. Motifs can be either sequential or structural. Motifs are represented as sequences when they represent repeated patterns in biological sequences. Motifs are structural when they represent patterns of conserved base pairs (e.g. RNA secondary structures) [1,2]. Knowing structural motifs in RNA leads to a better understanding of the mechanisms of action. Unlike DNA motifs, which are sequentially conserved, RNA motifs may share a common structure even in the case of low sequence similarity. Many algorithms have been devised to solve the structural motif discovery problem. However, due to the lack of a gold standard benchmark, little work has been done to evaluate their performance. A survey of different structural RNA motif discovery algorithms can be found in [3]. The structural motif discovery problem should not be confused with the two close problems: RNA structure prediction and RNA consensus structure prediction [4]. In the former, it is required to predict the secondary structure of a single RNA sequence. In general, the predicted structure minimizes the total free energy. While in the later, it is required to find a list of base pairs that can simultaneously be formed in a set of related RNA sequences. In this case, it is generally assumed that sequences are evolutionary related and share a similar overall fold. Evolutionary conservation information is utilized to improve the accuracy of structure prediction process. In this paper, we focus on structural motif discovery, where the goal is to discover repeated patterns in RNA secondary structures. The paper is organized as follows. In the following section, the motif discovery problem is formulated. Next, the basics of RNA secondary structures are presented including: common representations and free energy models that are used to evaluate the stability of an RNA structure. Then, we survey and classify motif discovery algorithms and discuss their advantages and limitations. We proceed by proposing a benchmark and a tool for evaluating the performance of motif discovery algorithms. Finally, our experimental setup and comparative results are presented and discussed.

## Motif discovery

The structural motif discovery problem can be formulated as follows: Given a set of RNA sequences *S*, it is required to find common structural motifs that are responsible for the function or regulation of the RNA sequences. In general the motif discovery problem is a challenging problem: The motif may not be present in all input sequences. In real situations, unrelated sequences may be included by mistake. Then, only a subset of the given sequences can share a common motif. In other situations the input sequences may be related, but there is no common single motif for all of them. This happens when sequences are functionally related but belong to different structural classes. For *N *sequences, there are 2^*N *^subsets of sequences that need to be examined to find a motif. Hundreds of algorithms and tools have been published to tackle motif discovery in the sequential case. However, less work has been done for structural motif discovery. The structural case is more difficult than the sequential case because motifs may share a common structure even if their underlying sequences are different.

## Preliminaries

### RNA secondary structures

RNA is found as a single strand where individual bases can bond with each other forming *base pairs *[5]. Bonding makes RNA fold into a structure called *secondary structure*. There are different rules for base paring [5]. A *Waston-Crick *base pair is formed when *A *bonds with *U *through a double hydrogen bond or when *G *bonds with *C *through a triple hydrogen bond. A *wobble base pair *is formed when G bonds with U by a single hydrogen bond. There are other pairing rules such as G-A and U-C pairs, but they are relatively rare [5]. RNA secondary structures can be *interacting *or *non-interacting*. In an interacting structure, base pairs may be formed from bases that belong to different strands (inter-molecular). In a non-interacting RNA structure, all base pairs are formed by bases in the same strand (intra-molecular).

### Definition: Secondary structure

Given an RNA sequence *R *= {*r*_1_*r*_2_...*r_n_*} of length *n*, the secondary structure *S *of *R *is a set of base pairs (*r_i_, r_j_*), where 1 ≤ *i *<*j *≤ *n*, that satisfies the following two criteria [5]:

(1) Each base is paired at most once.

(2) If (*r_i_, r_j_*), (*r_k_, r_l_*) ∈ *S*, then *i *<*k *<*j *⇔ *i *<*l *<*j*. This is called *the nested criterion*.

The criteria above may be rarely violated in RNA secondary structures. If the first one is not satisfied, then a *base triple *may happen. If the second is not satisfied, then a *pseudoknot *may exist [5].

### RNA representations

There are different ways to represent RNA secondary structures. Some popular RNA representations are:

#### Dot-bracket notation

**Figure 1:**
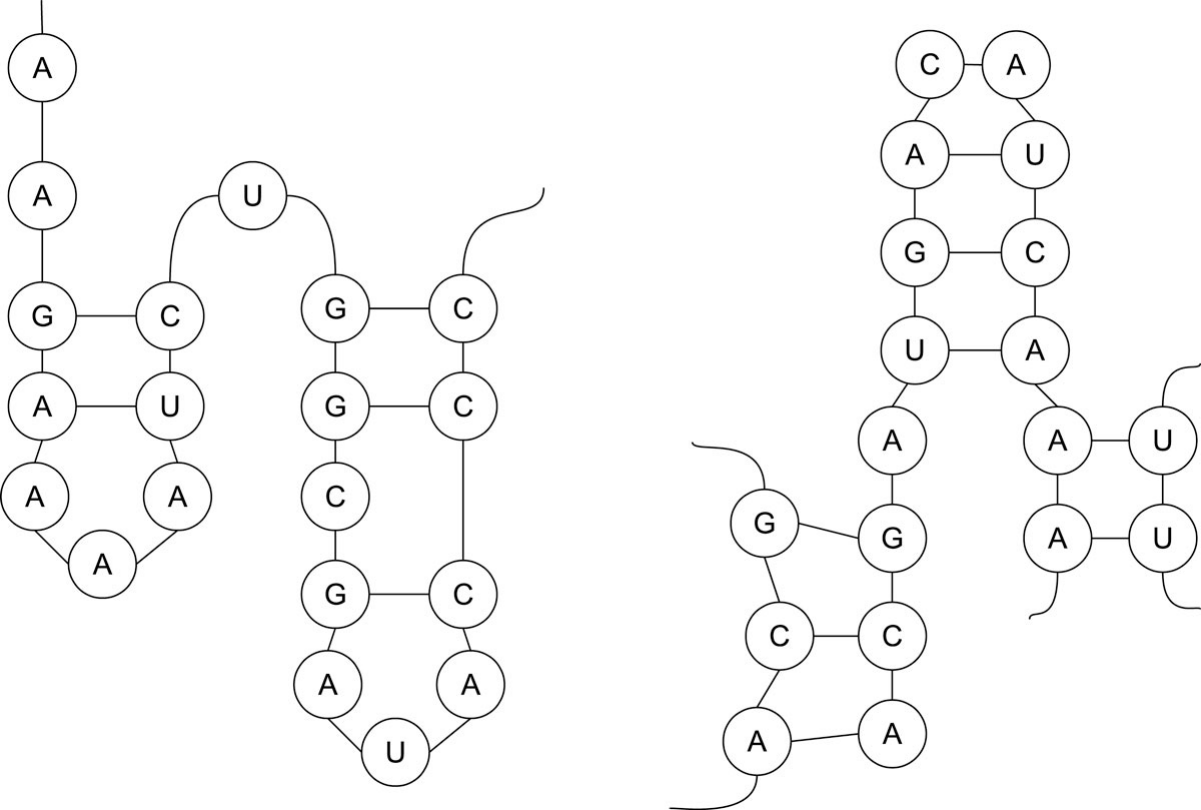
**RNA Secondary Structures**. RNA Secondary Structures. Left: non-interacting RNA (only intra-molecular base pairs). Right: interacting RNA (with inter-molecular base pairs).

**Figure 2:**
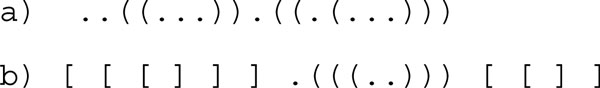
**Bracket notation**. Bracket notation for the structures in Figure 1.

RNA secondary structure can be represented as a string of length *n *over the alphabet Σ = {(, ., ), [,]}. This representation is known as *dot-bracket notation *(DBN), or *nested parenthesis*. Initially, proposed in [6], base pairs are represented using matched brackets. A base pair (*r_i_, r_j_*) is represented by an opening bracket at position *i *and a closing bracket at position *j*. Unpaired bases are represented by dots. Pseudoknots are represented using square or curly bracket. Sometimes square bracket are used to represent inter-molecular base pairs. An example of bracket notation representations of the secondary structures of Figure 1 are shown in Figure 2.

#### Planar graph

**Figure 3:**
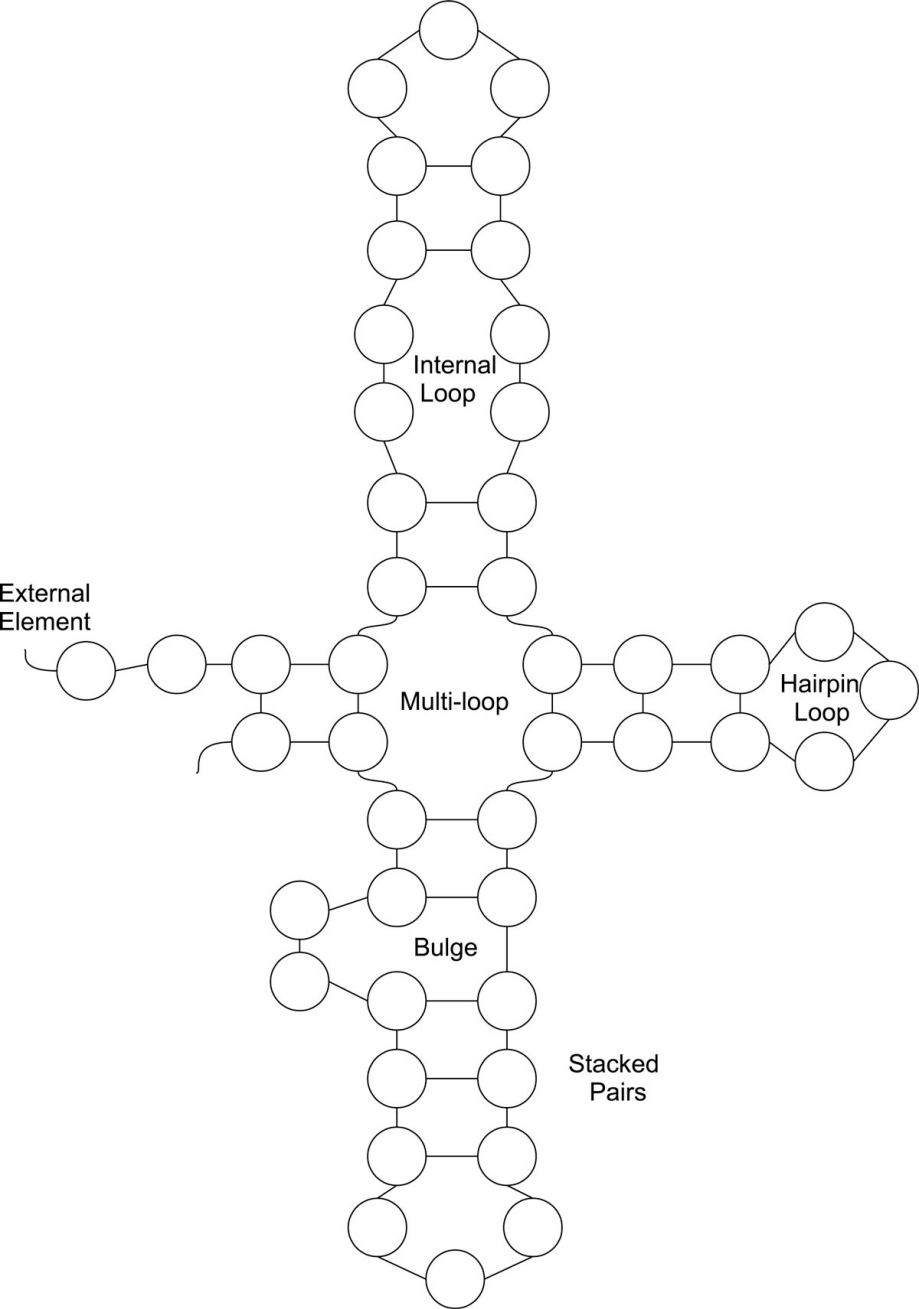
**Planar graph representation**. RNA various loop types in planar graph representation.

RNA secondary structure can be represented as a *planar graph *[6], also known as *bond representation*. The graph is a simple approximation of the RNA secondary structure in two dimensions. As shown in Figure 3, the planar graph may include the following different types of loops in the secondary structure:

• Hairpin loop: a loop that contains exactly one base pair.

• Staked pair: a set of consecutive base pairs.

• Internal loop: a loop with two base pairs and at least one unpaired base on each side of the loop.

• Bulge: Like an internal loop, a bulge has two base pairs but with only one side of the loop having an unpaired bases.

• Multi-loop: any loop with three or more base pairs.

• External base: any unpaired base not contained in a loop. A set of consecutive external bases form an *external element*.

#### RNA expression

RNA secondary structures can be represented as an expression of the following six types of terms [1]:

• H5 and H3: to represent the beginning and end of an intra-molecular stem.

• I5 and I3: to represent the beginning and end of an inter-molecular stem for interacting molecules.

• SS: to represented a single stranded region (unpaired).

• BR: to indicate a move between RNA sub-patterns in case of interacting patterns.

**Figure 4:**
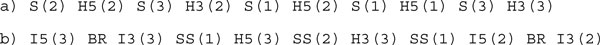
**RNA expression**. RNA expression for the structures in Figure 1.

Each term can be followed by tuple (*x, y*) to indicate the minimum and maximum length, respectively. Figure 4 shows the RNA expressions for the structures in Figure 1, where each term has a defined length.

#### Component-based representation

RNA secondary structures can be represented by components [1]. In this representation, a pattern can be defined by three parts: (1) its length, (2) the intra-molecular (INTRAM) component (3) the inter-molecular (INTERM) component if interacting. An interacting pattern consists of more than one sub-patterns. In general *P *= {*p*_1_, *p*_2_, ..., *p_m_*}, each *p_j _*= (*len_j_*, {*INTERM*_1_, *INTERM*_2_, ..., *INTERM_r_*}, {*INTRAM*_1_, *INTRAM*_2_, ..., *INTRAM_q_*}) for 1 <*j *≤ *m*. If the pattern is not interacting, when is *m *= 1, it will only have INTRAM component.

**Figure 5:**
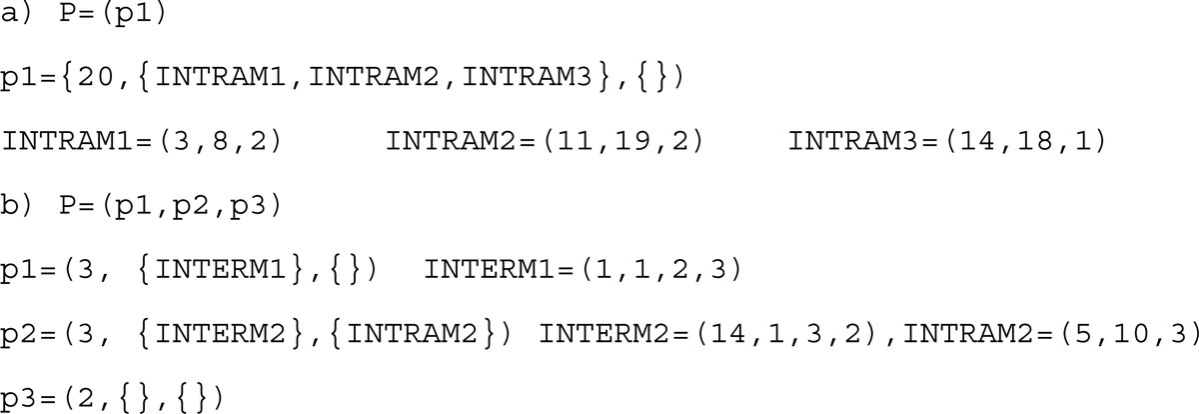
**Component-Based representation**. Component-Based representation.

Components are defined by the length of their opening and closing brackets and by their relative location in the pattern. The component-based representation of the structures in Figure 1 are shown in Figure 5.

#### Covariance model

**Figure 6:**
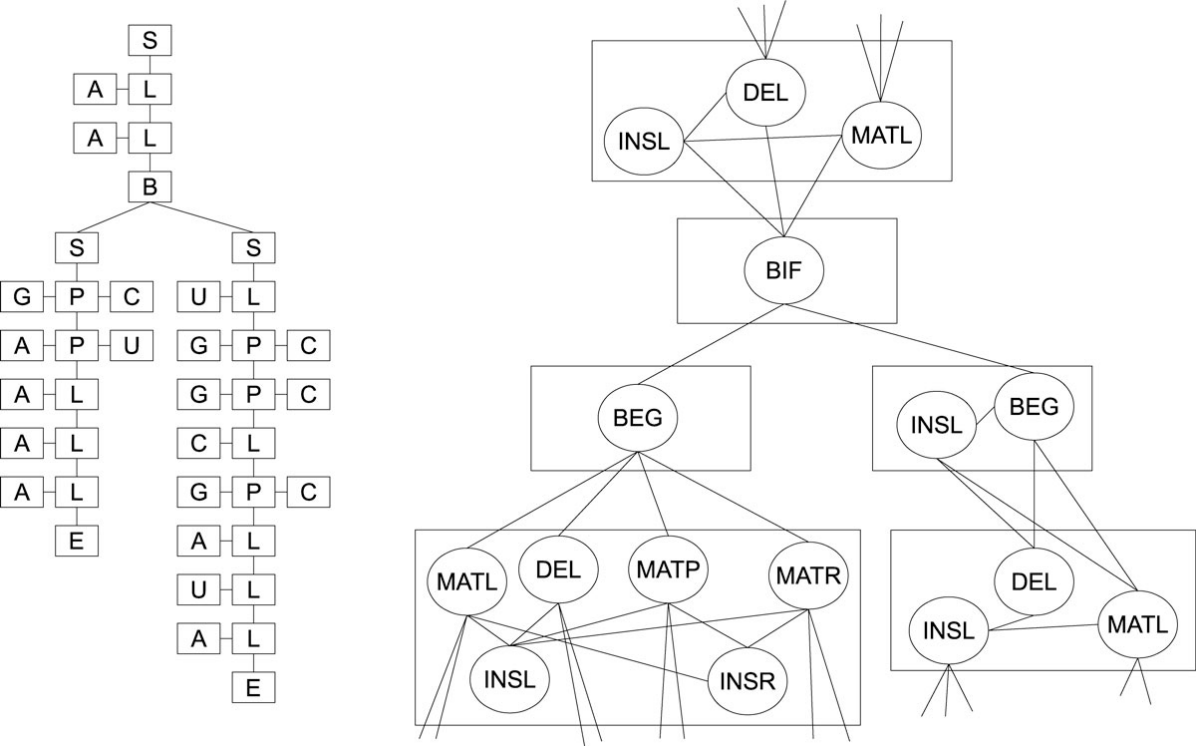
**Covariance model**. Covariance model: ordered binary tree (right) and the internal states (left) for parts of the non-interacting structure in Figure 1.

Covariance model (CM) [7] is a probabilistic model for describing a sequence alignment and a consensus secondary structure for a set of RNA sequences. It is represented as an ordered binary tree of different types of nodes: begin (S), pair (P), left singlet (L), right singlet (R), and bifurcation (B). Each node can have different number of states to allow insertions, deletions, and mismatches. CM is a generalization of the hidden Markov models with two additional states. The match pairwise state allows the emission of a pair of symbols, while the bifurcation state allows multiple helices. The CM works only for non-interacting RNA sequence from the 5' end to the 3' end. Figure 6 shows an example of a CM for the non-interacting RNA in Figure 1.

#### Connectivity table

RNA secondary structure can be represented as a table called *connectivity table *(CT). The table is formatted as follows:

• The first line shows the number of bases in the sequence and the name of the structure.

• Each following line provides information about one base pairs as follows:

- base index.

- base.

- next index.

- previous index.

- index of the base to which it is paired. Zero for no pairing.

- Natural numbering.

**Figure 7:**
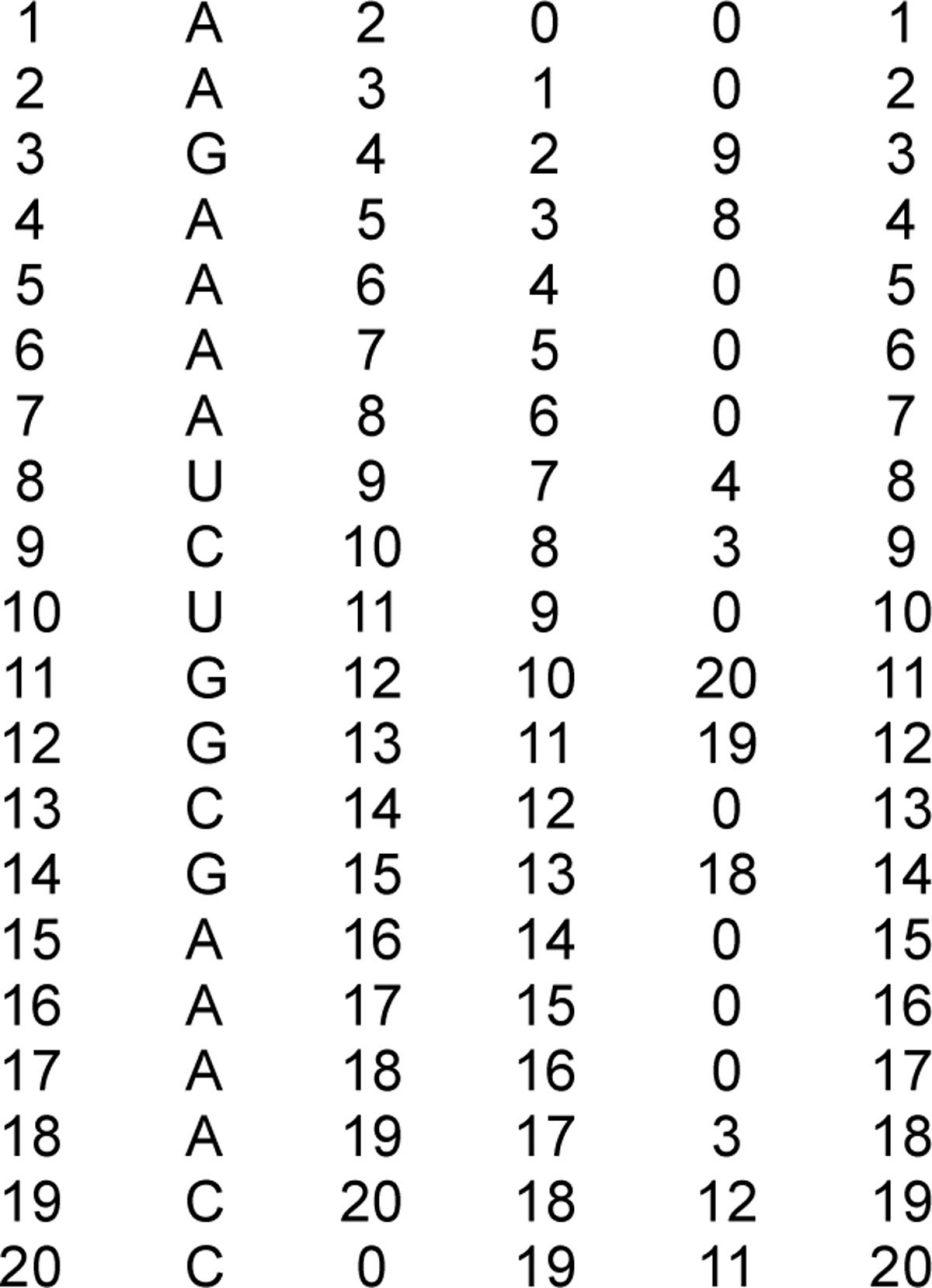
**Connectivity table**. Connectivity table for the non-interacting structure in Figure 1.

Figure 7 shows an example of connectivity table for the non-interacting RNA in Figure 1.

#### Arc representation

**Figure 8:**
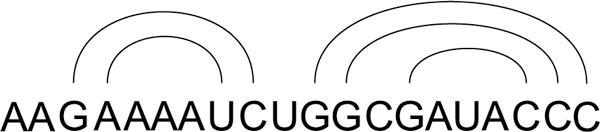
**Arc representation**. Arc representation.

In this representation, a base pair is represented as an arc connecting the two bonded bases. The secondary structure is a set of overlapping or parallel arcs [8]. An example of this representation is shown in Figure 8.

### Free energy models

The stability of RNA structures is determined by their *free energy*. Stable structures have the lowest free energy values. In this section, we discuss well-known models that are used to calculate the free energy.

#### Base pair energy model

The simplest energy model considers individual base pairs. It either maximizes the number of base pairs or uses the sum of free energies of individual Waston-Crick base pairs [9,10]. Waston-Crick free energy between base *i *and base *j *is denoted as *e*(*i, j*) and it is based on the type of bonded bases. The bond is called *internal*, if the bases are on the same RNA strand, otherwise it is called *external*. The energy functions for internal and external bonds may be equal. For any secondary structure *S*, the total energy is the sum of *e*(*i, j*) over all pairs as follows [11]:

$$E_{p}\left(S\right)=\underset{\left(i,j\right)\in S}{\sum }e\left(i,j\right)$$

Although this model is simple, it is known to be inaccurate [12]. The base pair maximization model does not yield biologically relevant structures. This is because: it ignores stacking base pairs, it dose not consider loop sizes, and it has no special scoring of multi-loops.

#### Stacked pair energies

The stacked pair model [13,14] assigns energy *E_s _*to a base pair (*i, j*) ∈ *S *if and only if (*i *+ 1, *j *− 1) ∈ *S*. The total energy under this model is given as follows:

$$E_{s}\left(S\right)=\underset{\left(i,j\right)\in S}{\sum }E_{s}S_{i+1,j-1}$$

Where *S*_*i*+1,*j*−1 _= 1 if the pair (*i *+ 1, *j *− 1) ∈ *S, S*_*i+1,j*−1 _= 0 otherwise. The total number of consecutive base pairs is called the *stacking size*. Single base pairs are not considered as stacks, so the staking size is at least 2.

#### Loop energy model

The loop energy model, also called the *nearest neighbor model *[15], considers the free energy of the different types of loops, including: free energy of externally interacting loops. Under this model, the free energy of a secondary structure is the sum of free energies of all of its component loops. This model appears to be more accurate especially for RNA molecules with length ≥ 150 [12]. The contribution of each loop type is determined as follows:

• Hairpin loop: the energy contribution of a hairpin loop is determined by two elements. First, the number of unpaired bases forming the loop. Second, the contribution of the *terminal mismatch*, which are the two bases adjacent to the closing base pair.

• Staked pair: the energy contribution of a stacked pair is determined by the type and order of base pairs.

• Bulge: the energy contribution of a bulge loop is determined by the length of the unpaired bases forming the bulge and the two closing base pairs.

• Internal loop: the energy contribution of an internal loop is determined by the length of the unpaired bases forming the loop and the four unpaired bases adjacent to the opening and closing base pairs.

• Multi-loop: the energy contribution of a multi-loop is a function of many factors, including: number of helices and the optimal configuration of free ends and terminal mismatches.

## Classification of RNA motif discovery algorithms

There are many approaches to solve the structural motif discovery problem. In this section, we classify the different approaches that solve this problem. Based on how the search space is explored, we classify the approaches into two main classes: *enumerative approaches *and *heuristics approaches*. In enumerative approaches, the search space is exhaustively explored in order to discover overrepresented motifs. Algorithms in this class can be further divided into: dynamic programming-based approaches (DP), data structures-based approaches (DS), and graph-based approaches (GB). On the other hand, in heuristic approaches, only promising regions of the search are explored. Examples of algorithms in this class include: *Expectation maximization *and *evolutionary algorithms*. In addition, there are other heuristics specifically designed to tackle the motif discovery problem.

### Enumerative approaches

#### • Dynamic programming approaches

Dynamic programming is a well-known algorithm design technique. It is based on the observation that within optimal solutions there exist optimal solutions to subproblems. Most motif discovery algorithms in this class rely on extending Sankoff's algorithm [16] for simultaneous folding and aligning RNA sequences. The algorithm combines the recurrences of sequence alignment and folding algorithms. Given two sequences, it finds two structures and an alignment such that the energy and alignment score are optimal. The two structures need to show the same kind of branching. This should also be reflected in the alignment where branches are aligned to each other.

FOLDALIGN [17-19] is a DP-based motif discovery tool for two sequences. The first implementation of FOLDALIGN [17] starts with pairwise alignments using a simple extension to Sankoff's algorithm. Instead of minimizing the total cost of the alignment and the energy of the structure, the proposed extension maximizes the alignment similarity and the number of base pairs that are formed in the two aligned sequences. The time complexity is reduced by not allowing branching structures. After the initial pairwise alignments are computed, they are aligned with all individual sequences. The algorithm proceeds by following a greedy (progressive) approach by aligning triplet alignments to every individual sequence and so on. In order to obtain an alignment of size *r*, only alignments between a single sequence and an *r *− 1 sequences are considered. After each round only the best 30 alignments are retained to generate *r *+ 1 alignments. For each round *r*, the few best alignments are taken and their score versus length is calculated. Scores are plotted as a function of *r*. The algorithm then stops at the round for which the rate of change decreases. Finally, the best five scored alignments from *r *and *r *− 1 sequences are considered. In subsequent implementations of FOLDALIGN [18,19], the basic algorithm was improved to deal with branched structures and to use a lightweight energy model.

SLASH [20] is an approach for the discovery of hairpin motifs in unaligned sequences based on two approaches: FOLDALIGN [21] and COVE [7]. FOLDALIGN is used to find local alignments in RNA sequences. Then COVE's model is trained on that alignment.

#### • Data structures-based approaches

Motif discovery algorithms can use data structures to accelerate the access and retrieval of words. Mauri and Pavesi [22] presented an algorithm using *affix trees *data structures for the discovery of hairpins, bulges and internal loops in RNA. An affix tree is a data structure that stores information about a given string and its reverse. The proposed algorithm has two inputs. First, a set of RNA sequences (denoted as *S*). Second, the minimum number of sequences in which a motif can appear (denoted as *q*). In addition, it has two optional inputs: the maximum value of loop size and the maximum number of unpaired bases that can form internal loops and bulges. The basic idea is to start by building an affix tree. From the affix tree all substrings of length *l *are identified. Substrings appearing in at least *q *sequences are then progressively expanded by adding one base pair or an unpaired base. The proposed algorithm terminates when motifs can not be expanded. Motifs are then evaluated to determine their free energy. A motif is reported only if its energy satisfies a given threshold.

Seed [23] uses another data structures, namely *suffix arrays*, for the discovery of structural motifs. In the algorithm one of the input sequences is considered as a seed. The data structure is then used to store the seed sequence and its reverse. The algorithm proceeds by listing all stems in the seed sequence. This is done by looking at complementary regions. Stems that appear in a number of sequences with frequency above a predefined threshold are kept for further processing. Then, base pairs in the listed motifs are replaced with the actual base pairs occurring in the seed sequence. Finally, multi-stem motifs are constructed by combining two motifs. Depending on the positions of the two motifs, they can be either nested in each other or put next to one another (adjacent relationship). The combination can result in different structures such as bulges, internal loops and multi-loops. In fact, the motifs capture both sequence and secondary structure information. They are ranked based on free energy.

#### • Graph-based approaches

comRNA [24] is a graph-based approach that can find pseudoknots. It starts by finding all possible stable stems of length *L *in each sequence using a branch and bound approach. The stability of a stem is evaluated based on stacking energy. Bulges and internal loops are not allowed at this stage. Next, the similarity between each stem pair is computed. Then, an *n *− *partite *undirected weighted connectivity graph is created. Nodes in the graph represent stems, while edge represent similarity values between them. The graph is partitioned to *n *parts, where each part consists of stems belonging in the same sequences. The final step is to find all conserved stems that are common to multiple sequences. In graph theory, this corresponds to finding a set of maximum *cliques*. A clique is a complete sub-graph where every node is connected to every other node. After that, a graph technique similar to topological sort is applied to find the best assemblies of stems. Finally, the highest ranking motifs are reported.

RNAmine [25] uses graph mining algorithm to discover motifs shared by a subset of different RNA sequences. An RNA sequence and its secondary structure is represented as a directed labeled graph, called *stem graph*. In a stem graph, a node denotes a stem and an edge denotes the relative positions of two stems. Initially, candidate stems are derived from McCaskill's algorithm [26] using Vienna package [27]. Stems shorter than a given threshold are discarded. The algorithm exhaustively enumerates stem patterns using a branch and bound algorithm. A pattern needs to satisfy three constraints: (1) A pattern that exist in at least *m *stem graphs, (2) A pattern should form a clique, and (3) It must not be a general pattern.

### Heuristic approaches

#### • Evolutionary algorithms

Evolutionary computation algorithms are based on biological evolution concepts such as natural selection, survival of the fittest, and reproduction to search for an optimal solution. The main components of evolutionary algorithms are: solution representation, fitness function, population initialization, selection, and reproduction operators. RNAGA [28] uses *genetic algorithms *(GA) to find common secondary structures in a set of homologues RNA sequences.

In RNAGA, GA is applied at different levels. A GA is applied to each sequence to get a set of stable structures. The fitness of a secondary structure is based on its free energy. Then, for each sequence, the resulting set of stable structures are evaluated to determine their conservation among different sequences. Using the conservation score as a measure of fitness, a GA is applied again to the set of stable structures. Finally, the GA terminates when maximum number of generations is reached.

GPRM [29] uses *genetic programming *(GP) to find structural motifs in RNA. The algorithm requires two sets of inputs: a set of co-regulated RNAs, called the positive set, and a set of randomly generated sequences, called the negative set. The initial population of motifs is generated based on a user-defined parameters: number of segment in a motif and the range of segment length. A segment is either Waston-Crick base pairs or unpaired bases. Individuals are evaluated based on *F-score*, which is a function of sensitivity and positive predictive values. The sets of positive and negative sequences are used in calculating the score.

GeRNAMo [30] also uses a GP to find RNA motifs. Unlike GPRM, where the initial motifs are randomly generated, GeRNAMo starts with a set of suboptimal secondary structures. First, a preprocessing step takes place where input sequences are broken into subsequeces ranging between a user-defined minimum and maximum lengths. Then for each subsequence, RNAsubopt [31] is used to find the suboptimal secondary structure based on thermodynamics evaluation. The output of RNAsubopt is then used to build an array that stores all the information of the suboptimal secondary structures. GP is then applied with a fitness function that favors motifs that are common to the majority of input sequences.

#### • Expectation maximization

Expectation maximization (EM) is a statistical method for parameter estimation in the case of incomplete data. CMfinder [32] is an RNA motif discovery algorithm that is based on *expectation maximization *(EM) and covariance model RNA representation. The algorithm starts by finding strong motif candidates. Those are the ones with more stable structure. For each input sequence, the minimum free energy of all subsequences is computed. Top ranking candidates are then chosen and compared at base level. The algorithm proceeds by constructing an initial alignment from similar candidates. The initial alignment is used as a seed for the EM algorithm. Then, EM takes place twice: First, to refine the initial alignment. Second, to scan each sequence looking for hits, where top hits are treated as candidates. Finally, motifs are merged to produce the final result.

RNApromo [33] is another EM-based method for identifying RNA motifs. It starts with a set of RNA sequences and a set of suggested secondary structures. The motif prediction algorithm is composed of two main parts. In the first part, several heuristics are employed to identify good motif candidates. In the second part, candidates are refined using an EM algorithm.

#### • Other heuristics

These are other algorithms that are specially designed to solve the motif discovery problem. RNAProfile [34] is composed of two steps. In the first step, a simple heuristic is used to extract a set of candidate regions from each sequence. Each region is associated with an optimal secondary structure. The heuristic then selects the regions whose predicted optimal secondary structures have exactly *h *hairpins. The second step involves grouping regions by progressively aligning profiles. Groups are scored based on the quality of the multiple alignment that is built from regions in the same group. The method requires one parameter, which is the number of hairpins in a motif. Table 1 summarizes different structural motif discovery algorithms.

**Table 1:** Structural motif discovery algorithms.

Tool	Class	Website
FOLDALIGN [17]	EN	[40]
Based on Sankoff's algorithm. It maximizes alignment similarity and number of base pairs formed in 2 aligned sequences.		
SLASH [20]	EN	NA
Uses FOLDALIGN to find local alignments in RNA sequences. Then COVE [41], to build a SCFG model from the local alignments.		
Mauri & Pavesi [22]	EN	NA
Uses Affix trees for the discovery of hairpins, bulges and internal loops in RNA. Substrings of certain length appearing in at least *q *sequences are found and expanded.		
Seed [23]	EN	[42]
Uses suffix arrays to induce motifs from the seed sequence. Data structures are used to store the seed sequence, its reverse, and the input sequences.		
comRNA [24]	EN	[43]
Uses an *n *− *partite *undirected weighted connectivity graph to represent stems and their similarity. The problem of finding motifs is mapped to finding a set of maximum *cliques*. A graph technique similar to topological sort is applied to find the best assemblies of stems.		
RNAmine [25]	EN	[44]
Uses a graph mining algorithm to find conserved stems.		
RNAGA [28]	HU	NA
Genetic algorithm is applied at different levels. First it is applied on each sequence to get a set of stable structures. Then it is applied again to the set of stable structures.		
GPRM [29]	HU	NA
Uses genetic programming. It requires two sets of inputs: a positive set and a negative set. Individuals are evaluated based on *F-score *and using the two input sets.		
GeRNAMo [30]	HU	NA
GeRNAMo applies genetic programming on the output of RNAsubopt.		
CMfinder [32]	HU	[45]
based on *expectation maximization *(EM) to simultaneously align and fold sequences using covariance model of RNA motifs.		
RNAProfile [34]	HU	[46]
Uses a heuristic to extract a set of candidate regions from each sequence. The second step involves grouping regions to find similar motifs.		
RNAPromo [33]	HU	[47]
The motif prediction algorithm initially looks for structural elements which are common to the input RNAs, and then employs an expectation maximization algorithm to refine the resulting probabilistic model.		

EN: enumerative and HU: heuristics.

## Advantages and limitations

There are many challenges facing the motif discovery algorithms: The ability to discover complex structures, including pseudoknots, the ability to deal with large datasets, scalability, and also the ability to distinguish biologically significant motifs. On one hand, according to the so far reviewed methods, dynamic programming based algorithms do not allow branching structures in order to reduce time complexity. Consequently, they are limited to very conserved stem-loops and are only suitable for small datasets. On the other hand, some data structure based-algorithms (e.g. Seed) and heuristics-based algorithms (e.g. CMfinder, RNApromo, and RNAProfile) can discover multi-loops. Graph theoretical algorithms (e.g. comRNA and RNAmine) can find pseudoknots and can discover multiple motifs from different families (e.g. RNAmine). However, they have non-polynomial time complexity because all algorithms are mapped to the clique finding algorithm, which is well-known to be NP-complete [35]. Evolutionary algorithms can deal with more complex secondary structures. Unfortunately, they are computationally demanding for large datasets and they may produce different results when run multiple times on the same data. Table 2 summarizes the advantages and limitations of the different motif discovery approaches.

**Table 2:** Advantages and limitations of the surveyed motif discovery approaches.

Approach	Structures	Pseudoknots	Scalablity
Dynamic Programming	stems	no	no
Data Structures-Based	multi loops	no	yes
Graph-Based	complex	yes	no
Evolutionary Algorithms	complex	yes	no
Expectation maximization	complex	yes	no

## Proposed benchmarks

Motivated by the lack of a 'gold standard' benchmark, we propose a benchmark that can be used to assess the performance of structural motif discovery tools. The benchmark is specifically designed to highlight the different challenges of the motif discovery problem. The complexity of the RNA secondary structures depends on the number loops and the type of loops. Changing these two parameters can vary the complexity of the structure. Thus, based on the complexity of the allowed RNA secondary structures, the benchmark can be divided into the following datasets, ranging from low complexity to high complexity structures:

• Ref.1 contains three RNA families: Iron response element I (IRE), Histone 3' UTR stem-loop (Histone3), and Selenocysteine insertion sequence 1 (SECIS I). These families have simple secondary structures that are composed of a few number of loops and loop types (1 to 2).

• Ref.2 contains three RNA families: FMN riboswitch, glmS ribozyme, and Lysine riboswitch. These families have more complex secondary structures that are composed of a higher number of loops (5 to 7) and loop types (2 to 3).

• Ref.3 contains three RNA families: Enteroviral 3' UTR element (Entero_OriR), Metazoan signal recognition particle RNA (Metazoa_SRP), and Bacterial RNase P class B (RNaseP_bact_b). These families have complex secondary structures that are composed of a large number of loops (8-17) and loop types (4-5).

**Figure 9:**
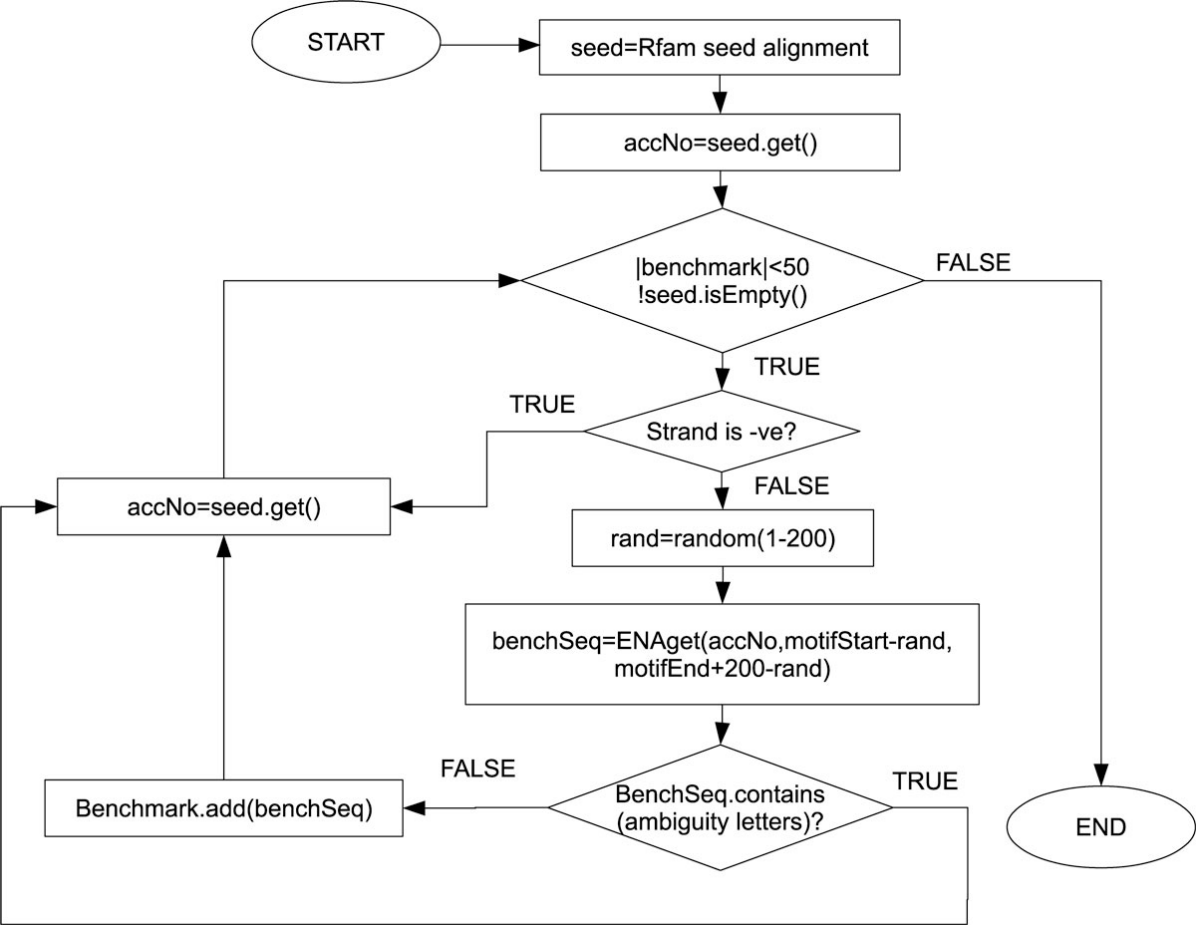
**Benchmark generation**. Benchmark generation.

The datasets were retrieved from the Rfam database [36,37]. Figure 9 describes the benchmark generation process. For each family, seed alignments were considered as the motif. From the seed sequences, all sequences with motifs appearing in the reverse complement were removed. Then at most 50 accession numbers, which are unique identifiers assigned to each biological sequence, were chosen randomly from the remaining sequences. For each accession number, the subsequence corresponding to the motif with 200 nucleotides of flanking regions was retrieved directly from the European Nucleotide Archive at the European Bioinformatics Institute [38]. Flanking regions were randomly distributed between the 5' and 3' ends. Retrieved subsequences containing ambiguity letters were discarded. The generation of the benchmark was conducted using a Java tool [39] specifically written for this task. The tool can also generate benchmark from a flat file provided by the user in FASTA format. Scalability can be measured by changing the length of flanking regions.

## Proposed measurement tool

To evaluate the performance of the available motif discovery tools, we developed a tool [39] to measure their performance in terms of sensitivity (Sn), specificity (Sp), and positive predictive value (PPV). The tool computes statistics for the discovered structures at the base pair level relative to the corresponding structures in the Rfam seed alignments. A discovered base pair (*i, j*) is considered true positive (TP), if it is identical to the known pair or it is shifted by one nucleotide on one side only. In other words, if (*i, j*), (*i *+ 1, *j*), (*i *− 1, *j*), (*i, j *+ 1), *or *(*i, j *− 1) is discovered [4].

**Figure 10:**
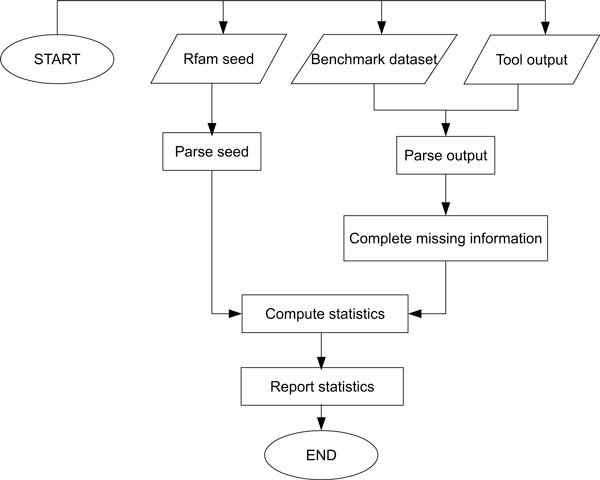
**Measurement tool**. Measurement tool.

As shown in Figure 10, the tool takes as an input: Rfam seed alignment, benchmark dataset, and the output of the tested tool. Each motif discovery tool has its own output format. For this reason, our measurement tool is designed to take this into consideration. The tool parses the output of each motif discovery tool and maps the results to an object of class Motif, which is then compared to the known motif represented by the Rfam seed alignment. The tool then uses the benchmark dataset to add any missing information in the output. RNAProfile, for example, outputs a motif instance in each input sequence without indicating the start and end positions of the motif instance. Other tools do not give the original start and end positions of the motif, instead they report positions relative to the start of the input sequence. Knowing the original positions is important for the sake of comparison with known structures in the Rfam seed alignment. Our tool is able to extract these types of missing information using the benchmark datasets.

## Experimental setup

To the best of our knowledge, this is the first comparison between tools designed for the purpose of structural motif discovery. For our experiment, we focus on the tools that discover structural motifs in more than two input sequences, where the input sequences are different, but share a common RNA structure. Among the surveyed tools, we were able to obtain six: CMfinder [32], RNAProfile [34], RNAmine [25], comRNA [24], RNAPromo [33], and Seed [23]. Our first observation is that the use of structural discovery tools often requires knowledge of the Unix operating system. Except for RNAmine, all the tools were available as a source code that needs to be unpacked and compiled. Difficulties arise when the tool depends on some old libraries. In addition, some tools require the determination of many parameters, especially Seed.

We were able to successfully compile and test the tools using a 64-bit Linux based operating system with 32 GB of RAM. All the tools were required to output up to ten motifs. The motifs were ranked according to the score used for each tool and the best scoring motif was compared to the known structure. The results were summarized by averaging statistics over secondary structures. Running the tools with default parameters was not always a good choice, so we had to tune parameters. The parameters used for each tool and each benchmark dataset are as follows:

• CMfinder: for Ref.1, all parameters were set to the default values. For Ref.2, the number of stem-loops was set to two, the minimum and maximum lengths of a motif were set to: 100 and 200, respectively. The same parameters were used for Ref.3, except for the maximum length of a motif was set to 400 for the families RNaseP_bact_b and Metazoa_SRP. The predictions of Ref.2 and Ref.3 were post processed using a tool provided by CMfinder [32] where a heuristic is used to combine multiple motifs.

• RNAProfile: for families Entero_OriR, FMN riboswitch, Lysine riboswitch, and glmS ribozyme the number of hairpins was set to 4 and the minimum and maximum length of a motif was set to 100 and 200, respectively. For the families RNaseP_bact_b and Metazoa_SRP, the the number of hairpins was set to 5 and the minimum and maximum length of a motif was set to 300 and 400 respectively. The motifs were scored based on the average alignment score for each structure.

• RNAmine: for Ref.1, the minimum size of stem candidate was set to 1. For the two families in Ref.3: RNaseP_bact_b and Metazoa_SRP, the maximum motif size was set to 600 and 400, respectively.

• comRNA: for all the datasets, the tool was run with the same parameter values. The minimum percentage of sequences in which a common structure should occur was set to 0.7. The maximum time allowed for structure finding with a certain stem similarity score threshold was set to 20 minutes. The maximum pattern search time allowed for RNAMOT was set to 20 seconds.

• RNApromo: for Ref.1, all parameters were set to the default values. In Ref.2 and family Entero_OriR of Ref.3 the minimum and maximum lengths of a motif were set to 100 and 250 respectively. For the remaining two Ref.3 families, the maximum length was set to 400.

• Seed: Setting parameters for Seed was difficult. Without proper parameter values, Seed either fills the entire hard disk with motifs or fails to discover motifs. For Ref.1, stem minimum length was set to 3 with maximum distance between the start and end of a stem set to 30 for family IRE. For the other two families in Ref.1, the stem minimum length was set to 6 and a maximum number of stems was equal to 1. For Ref.2, the fraction of input sequences having a motif (min-support) was set to 0.7. The stem minimum length and the maximum number of stems in a motif were set to 3 and 4, respectively. For Ref.3, the maximum separation was set to 10, minimum support to 0.8, stem minimum length to 3, and maximum number of stems to 5. For all the datasets, no mismatches were allowed. The motifs were scored based on the average minimum free energy for each structure.

## Comparative results

In this section, we show and discuss the results obtained when testing the available tools using our proposed benchmark. Results are obtained for sensitivity (Sn), positive predictive value (PPV) and specificity (Sp). All tools are able to discover motifs in all benchmark datasets, except for comRNA, which is able to discover motifs in two families only, RNaseP_bact_b and glmS ribozyme. Part of this could be due to using the defaults for the minimum similarity score between two stems. In addition, the threshold on the percentage of sequences in which a common structure should appear is higher than the threshold used in [24,32].

**Figure 11:**
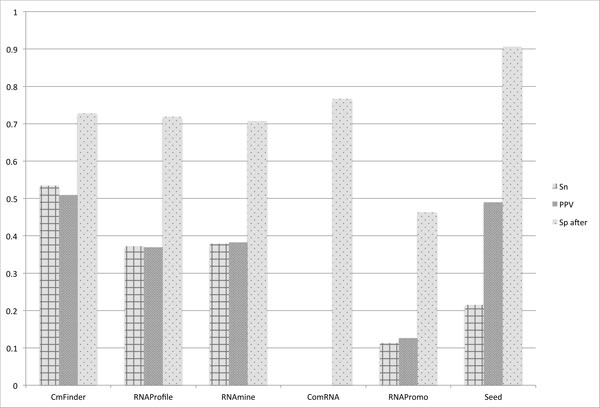
**Measurements averaged over all benchmark datasets**. Sensitivity (Sn), Positive Predictive Value (PPV), and Specificity (Sp) averaged over all benchmark datasets.

Figure 11 summarizes the results over the three accuracy measures averaged over all benchmark datasets. The figure shows that in general the accuracy of motif discovery tools is low. The average Sn of current tools over all datasets is 0.27 and average PPV is 0.31. CMfinder performs better than all the other tools in terms of Sn and PPV. While Seed has the best Sp.

**Figure 12:**
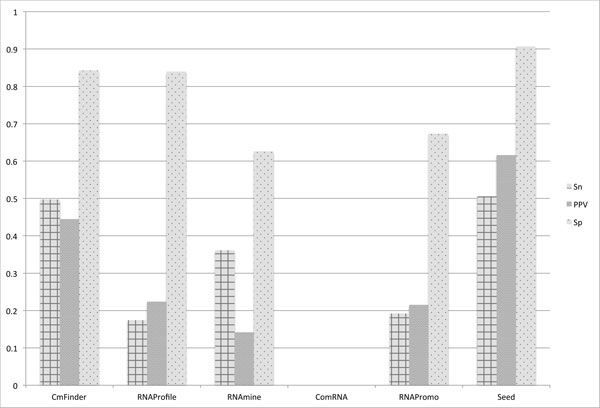
**Measurements averaged over all simple datasets, Ref**.1. Sensitivity (Sn), Positive Predictive Value (PPV), and Specificity (Sp) averaged over all simple datasets, Ref.1.

**Figure 13:**
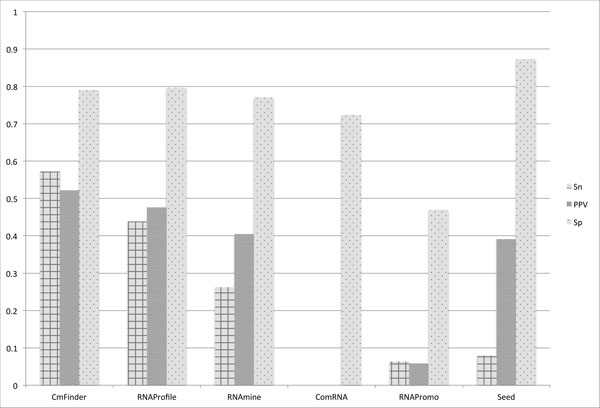
**Measurements averaged over all datasets in Ref**.2. Sensitivity (Sn), Positive Predictive Value (PPV), and Specificity (Sp) averaged over all more complex datasets, Ref.2.

The tools were evaluated based on the complexity of the RNA structures. Figure 12, 13, and 14 present the results for Sn, PPV, and Sp respectively for the three different levels of structure complexity. As for the simple structures, Ref.1, Seed and CMfinder have the best accuracy in terms of all measures. This indicates that the two tools are able to discover more simple motifs than the other tools. The accuracy of the rest of the tools is much lower.

**Figure 14:**
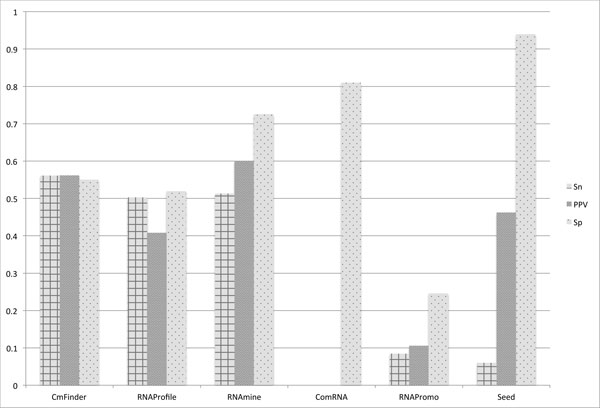
**Measurements averaged over all datasets in Ref**.3. Sensitivity (Sn), Positive Predictive Value (PPV), and Specificity (Sp) averaged for the most complex datasets, Ref.3.

For the more complex structures, Ref.2, CMfinder appears to have the best accuracy in terms of Sn and PPV, while the accuracy of RNAPromo and Seed in terms of Sn is considerably low.

As for the most complex structures, Ref.3, CMfinder has the best accuracy values in terms of Sn. The performance of RNAmine and RNAprofile is comparable to CMfinder. In terms of PPV, RNAmine shows the best performance. As for Sp, Seed outperforms all the other tools for the complex structures, Ref.2 and Ref.3.

CMfinder is the only tool that performs best for all the complexity levels of RNA structures. This can be attributed to the combination of rich initialization step and EM for refining profiles. The initial candidates in CMfinder are stable structures derived using Vienna package and are not limited to stem loops.

Seed performs well on simple structures. However, the performance decreases by 80%, in terms of Sn, on datasets with complex structure. This indicates that the simple enumeration of stem loops works well for discovering simple structure. The later combination of stem loops does not seem to yield real complex structures. However, the authors showed that the accuracy can be improved when the discovered motifs are used as constraints for MFOLD.

As for RNAProfile, in terms of Sn, it appears to perform better on complex structures. However, the accuracy decreases by 65% on benchmark datasets with simple structure. RNAProfile follows a progressive alignment approach. The degradation in accuracy could be due to the fact the dataset of complex structures have more sequence identity than the dataset of simple structures. The figures suggest a complementary behavior among tools.

The values obtained for Sn, PPV, and Sp are further analyzed using statistical significance tests. Table 3 shows the results of ANOVA test with *α *= 5%. The table indicates a statistical significance of the comparative results that are presented in this study.

**Table 3:** Results of statistical significance test with *α *= %5.

Measure	F-calc	p-value	F-crit
Sn	4.68294	0.0034	2.60597
PPV	4.08559	0.0072	2.60597
Sp	6.15544	0.00024	2.44343

**Figure 15:**
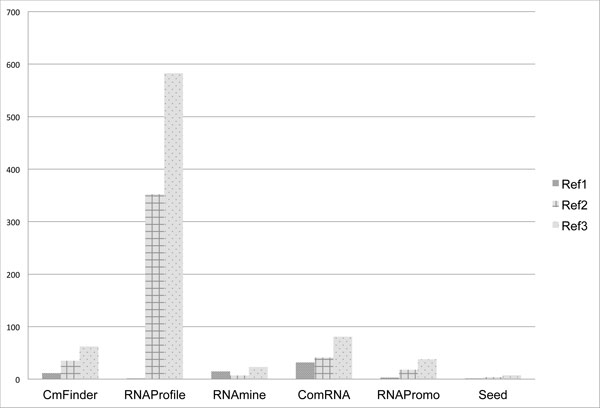
**Running time**. Average running time (linux real time converted to minutes).

Table 4 and Figure 15 show the running time in minutes per dataset type. For all the runs, the CPU time (real) was reported using the Linux time command. Regardless of the structure complexity, Seed has the least time requirements. This could be due to the use of data structures and a high minimum support threshold. For the complex structures in Ref.2 and Ref.3, RNAprofile was the most time consuming among all tools. This is because the algorithm processes regions of all possible lengths in each input sequence.

**Table 4:** Average running time (linux real time converted to minutes).

	CmFinder	RNAProfile	RNAmine	ComRNA	RNAPromo	Seed
Ref1 1	1.35	0.61	14.90	31.83	2.88	1.28
Ref2	34.87	351.99	7.14	41.28	18.12	3.81
Ref3	62.19	582.78	23.27	80.79	37.97	7.44

## Conclusion

In this paper, we surveyed and classified different algorithms that solve the structural motif discovery problem. We explored their strengths and weaknesses. Motivated by the lack of a gold standard to benchmark structural motif discovery tools, we proposed a benchmark datasets based on the complexity of the RNA structures. The benchmark can be used to evaluate different structural motif discovery approaches. In addition, we presented our measurement tool that can be used by other developers to compute the accuracy measures for their own approaches. We used the proposed benchmark dataset to evaluate six structural motif discovery algorithms. The obtained results show that the accuracy levels are generally low. CMfinder performs well regardless of the complexity of RNA structures. Some tools are more suitable to discover simple structure, such as Seed, while others are better for the discovery of complex structures, such as RNAmine and RNAProfile. This suggests a complementary behaviour among tools. Combining the results from different tools or from different runs of the same tool, may improve the accuracy of structure motif discovery. This has shown to be useful in motif discovery in the sequential case.

The performance evaluation of the tools were based on statistical measures. For each tool, the discovered motifs were compared to known motifs. The problem was when a new motif was (computationally) discovered; determining its biological significance remained a big challenge. Different tools relied on different objective functions to score motifs; this includes: thermodynamic stability, alignment score, and probabilistic measures. However, existing objective functions were unable to captures the hidden features of biologically relevant motifs.

## Competing interests

The authors declare that they have no competing interests.

## Authors' contributions

Ghada Badr conceived, designed, and coordinated the study, helped in drafting of the manuscript, and critically revised the final manuscript. Isra Al-Turaiki designed the benchmark, developed the measurement tool, carried out motif discovery tools testing, and helped in drafting the manuscript. Hassan Mathkour participated in the coordination of the study. All authors participated in analysis and interpretation of results. All authors read and approved the final manuscript.

## Declarations

Publication of this article was funded by the authors.

This article has been published as part of *BMC Bioinformatics *Volume 14 Supplement 9, 2013: Selected articles from the 8th International Symposium on Bioinformatics Research and Applications (ISBRA'12). The full contents of the supplement are available online at http://www.biomedcentral.com/bmcbioinformatics/supplements/14/S9.
